# Manipulating neutrophil degranulation as a bacterial virulence strategy

**DOI:** 10.1371/journal.ppat.1009054

**Published:** 2020-12-10

**Authors:** Kara R. Eichelberger, William E. Goldman

**Affiliations:** Department of Microbiology and Immunology, The University of North Carolina at Chapel Hill, Chapel Hill, North Carolina, United States of America; Duke University School of Medicine, UNITED STATES

## Neutrophil degranulation during bacterial infection

Armed with an arsenal of antimicrobial mechanisms, neutrophils are among the first innate immune cells recruited to the site of bacterial infection. Neutrophils utilize both oxidative and non-oxidative strategies to kill invading microorganisms. Components for non-oxidative killing include antimicrobial proteases that are packaged within intracellular vesicles (“granules”). Neutrophil granules are preformed vesicles of a defined composition that are released in a regulated manner [1]. The process by which neutrophils mobilize granules is called degranulation. Degranulation can occur at the plasma membrane for extracellular release (killing extracellular microorganisms) or to the phagosome for intracellular delivery (killing intracellular microorganisms) [1]. Extracellular degranulation is a double-edged sword of neutrophil antimicrobial function: The antimicrobials contained within granules can kill bacteria, but excessive degranulation can damage host tissue [2].

Neutrophil granules can be broadly categorized into 4 main types: secretory, tertiary, secondary, and primary. The release of each granule type occurs sequentially, with secretory granules released readily throughout the neutrophil life span to replenish cell surface receptors and primary granules requiring the greatest stimulus for release [1]. These granule types are classified based on the specific proteins contained within the lumen or in the membrane of each vesicle [3,4]. Secretory granules contain plasma proteins and Fc and complement receptors. The contents of tertiary granules include matrix metalloproteases such as matrix metallopeptidase 9 (MMP9). Secondary granules contain proteins such as lysozyme, pre-cathelicidin, and lactoferrin. Primary granules contain the most pro-inflammatory and antimicrobial proteins, such as myeloperoxidase, defensins, elastase, and azurocidin [5]. Additionally, during a normal neutrophil degranulation response, most of primary and secondary granule release is directed to the phagosome as a mechanism for minimizing damage to host tissue [6]. The extracellular release of each granule type can be assessed experimentally by detecting amounts of granule proteins present in supernatants by western blotting or ELISA or by quantifying the display of specific membrane-bound proteins (such as CD66b for secondary granules and CD63 for primary granules) on the surface of the neutrophil by flow cytometry or immunofluorescence microscopy.

**Fig 1:**
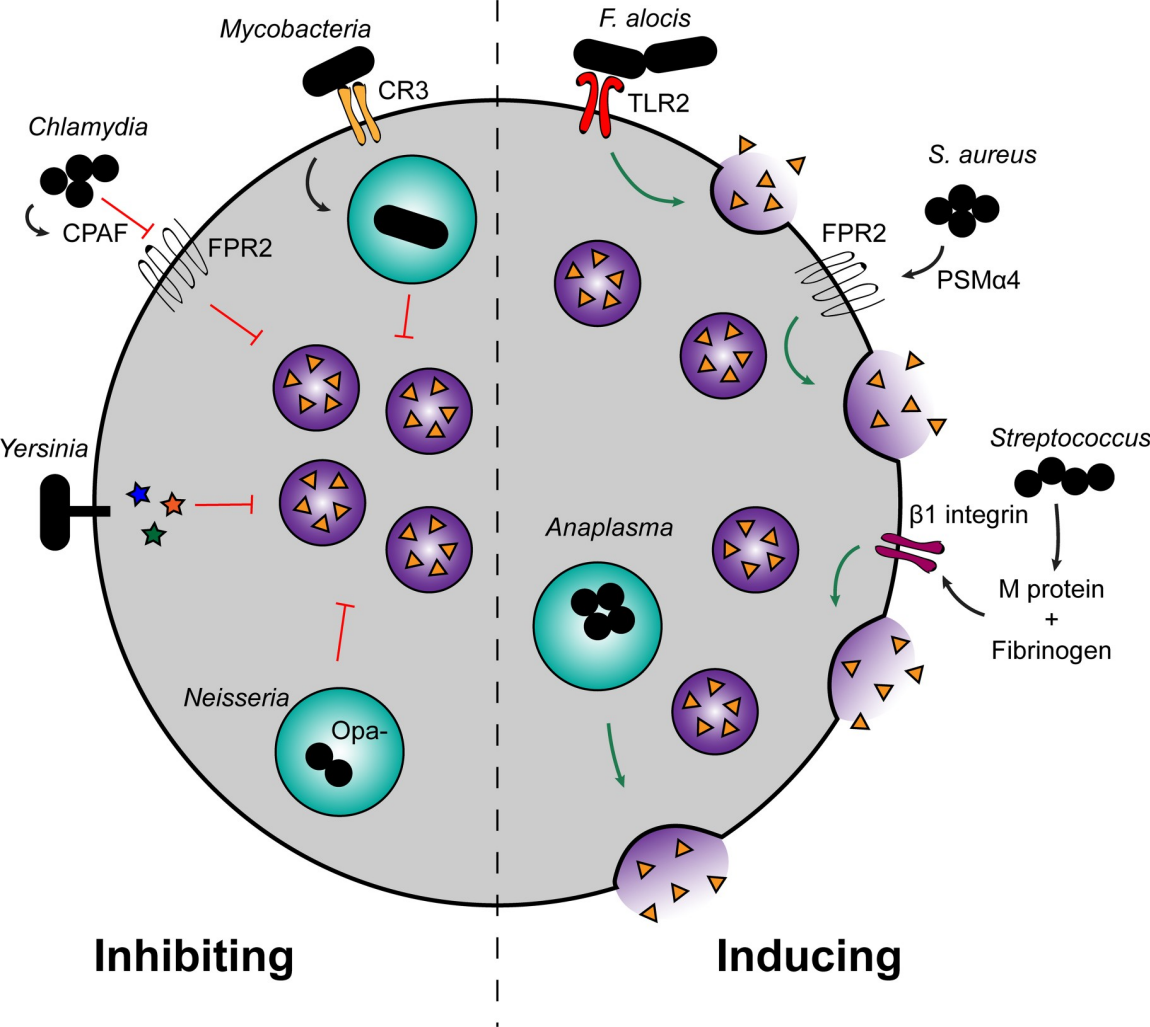
Bacterial modulation of neutrophil degranulation. Several pathogens either induce or inhibit neutrophil degranulation to promote infection. Uptake of *Mycobacteria* by CR3 passively prevents fusion of granules with the phagosome. *Chlamydia* produces CPAF, which cleaves FPR2 to inhibit degranulation. *Yersinia* injects effectors via the type III secretion system to inhibit degranulation, and *Neisseria* that does not display Opa reduces fusion of neutrophil granules with the phagosome. On the other hand, *Filifactor alocis* induces TLR2 signaling, triggering degranulation. *Staphylococcus aureus* produces PSMs, of which PSMα4 stimulates degranulation through FPR2. *Streptococcal* species that produce M protein also induce neutrophil degranulation by complexing with fibrinogen and binding β1 integrins. Finally, *Anaplasma phagocytophilum* induces neutrophil degranulation, although the exact mechanism remains unknown. CPAF, chlamydial protease-like activity factor; CR3, complement receptor 3; FPR2, N-formyl peptide receptor 2; Opa, opacity-associated protein; PSM, phenol-soluble modulin; TLR2, Toll-like receptor 2.

Several bacterial pathogens are known to manipulate neutrophil degranulation as a virulence strategy (Fig 1). By disrupting, dysregulating, or inducing excessive neutrophil degranulation, bacteria can skew the protective effects of neutrophil degranulation in a way that ultimately benefits the pathogen and worsens disease. Understanding the mechanisms by which bacteria alter neutrophil degranulation can provide greater insight into bacterial pathogenesis as well as advance our understanding of neutrophil vesicle trafficking.

## Mechanisms of inhibiting neutrophil degranulation

Inhibiting neutrophil degranulation can promote bacterial survival by preventing the targeting of antimicrobial proteins either intracellularly to the phagosome or extracellularly to the plasma membrane. Such inhibition would allow intracellular pathogens to utilize neutrophils as an infectious niche or allow extracellular pathogens unrestricted growth and/or dissemination. One strategy for inhibiting neutrophil degranulation is through targeting neutrophil cell surface receptors. Stimulation of integrins, G protein-coupled receptors, or L-selectin at the neutrophil surface can lead to intracellular signaling events that trigger increases in intracellular calcium levels, which induces granule exocytosis [1,5]. *Chlamydia trachomatis* produces the protease chlamydial protease-like activity factor (CPAF), which is released extracellularly as *Chlamydia-*infected epithelial cells lyse, and CPAF cleaves N-formyl peptide receptor 2 (FPR2) from the neutrophil cell surface [7]. FPR2 is a G protein-coupled receptor that signals through phosphoinositide 3-kinase (PI3K) to induce calcium flux and cytoskeletal rearrangements that trigger neutrophil degranulation. CPAF-mediated cleavage of FPR2 prevents neutrophil degranulation as measured by CD11b (contained in multiple granule types) and CD35 (secretory granules), as well as respiratory burst and neutrophil extracellular trap (NET) production [7]. The authors propose that this CPAF-mediated inhibition of neutrophil function via FPR2 targeting allows *Chlamydia* to escape neutrophil killing when the bacteria are released from infected epithelial cells.

*Mycobacteria* also target cell surface receptors to inhibit neutrophil degranulation, albeit by a passive mechanism. *Mycobacterium smegmatis* engages neutrophils through complement receptor 3 (CR3), which induces phagocytosis but prevents the downstream fusion of primary granules with the phagosome [8]. This inhibition occurs whether *M*. *smegmatis* are live or heat killed, and opsonizing the bacteria to induce phagocytosis through the Fc gamma receptor (FcγR) instead of CR3 stimulates granule fusion [8]. Cougoule and colleagues speculated that neutrophil granule fusion downstream of CR3 is triggered following clustering of the receptor, whereas opsonization is sufficient to trigger degranulation via FcγR. Early during infection, unicellular *M*. *smegmatis* is able to bypass this CR3-mediated activation by engaging with a single receptor [8]. *Neisseria gonorrhoeae* also inhibits granule fusion with the phagosome through selective targeting of neutrophil surface receptors. *N*. *gonorrhoeae* that display opacity-associated (Opa) proteins on their surface bind carcinoembryonic antigen-related cell adhesion molecules (CEACAMs), which triggers Src kinase signaling required for primary granule fusion with the phagosome [9]. Opa− *N*. *gonorrhoeae*-containing phagosomes have less primary granule fusion and greater intracellular survival [9]. Lastly, intracellular *Streptococcus pyogenes* inhibits granule fusion with the phagosome as well. Staali and colleagues determined that fewer primary granules fuse with phagosomes that contain *S*. *pyogenes*, and this inhibition is dependent on the production of M protein or M-like proteins [10]. However, secondary granule fusion with the phagosome is not inhibited by *S*. *pyogenes* M protein [10]. M protein is a bacterial surface protein, and therefore, it may also mediate selective engagement of neutrophil cell surface receptors without triggering downstream granule fusion with the phagosome, similar to *Mycobacteria* and *N*. *gonorrhoeae*.

Bacterial pathogens can also inhibit neutrophil degranulation downstream of cell surface receptors by blocking cell signaling pathways. *Yersinia* spp. encode a type III secretion system that injects effectors (*Yersinia* outer proteins [Yops]) directly into the neutrophil cytoplasm. Two effectors, YopE and YopH, cooperate to inhibit secondary granule release from neutrophils in *Yersinia pseudotuberculosis* [11] and primary and secondary granule release in *Yersinia pestis* [12,13]. Through the use of various chemical inhibitors, Taheri and colleagues demonstrated that *Y*. *pseudotuberculosis* inhibits secondary granule release through YopE-/YopH-mediated effects on calcium flux, actin dynamics, and PI3K signaling [11]. Additionally, *Y*. *pestis* blocks primary granule release from neutrophils through YopE inhibition of Rac signaling and YopH inhibition of calcium flux [12]. Minor roles for the effectors YpkA and YopJ, particularly in the absence of YopE or YopH, have been proposed in inhibiting neutrophil degranulation, but the precise neutrophil signaling pathways targeted by YpkA and YopJ remain to be determined [13].

## Mechanisms of inducing neutrophil degranulation

While several pathogens inhibit neutrophil degranulation to promote survival, there are instances of bacteria that induce granule exocytosis. The mechanism of this induction often results from virulence factor or bacterial engagement of specific neutrophil surface receptors. *Staphylococcus aureus* secretes a variety of toxins, including phenol-soluble modulins (PSMs). Lin and colleagues determined that PSMα4 activates FPR2 to trigger degranulation [14], and this is the same receptor cleaved by *C*. *trachomatis* to inhibit degranulation, as discussed previously [7]. The degranulating neutrophils release heparin-binding protein, which induces vascular leakage in vivo that contributes to the severity of *S*. *aureus* infection [14]. *S*. *pyogenes* induces neutrophil degranulation and the release of heparin-binding protein through M protein, which complexes with fibrinogen and binds β1 integrins to trigger neutrophil degranulation [15]. As discussed previously, M protein has also been shown to prevent fusion of primary granules with the *Streptococcus-*containing phagosomes in neutrophils [10]. The opposing effects of M protein may depend on the location of *S*. *pyogenes* in reference to the neutrophil (intracellular versus extracellular bacteria). In addition to G protein-coupled receptors and integrins, engagement of Toll-like receptors (TLRs) can also trigger neutrophil degranulation. The oral pathogen *Filifactor alocis* induces extracellular degranulation through engagement of TLR2, which triggers p38 mitogen-activated protein kinase (MAPK) activation to release secondary, but not primary, granules [16].

Additional cell surface receptors are also engaged by various pathogens to stimulate degranulation during infection. *Anaplasma phagocytophilum* is an obligate intracellular pathogen that induces neutrophil extracellular degranulation of both primary and secondary granules [17]. It was recently discovered that *A*. *phagocytophilum* uses an adhesin, Asp1, to bind protein disulfide isomerase (PDI) on the neutrophil surface, promoting invasion [18]. As PDI is contained within neutrophil secondary and tertiary granules [19], it is possible that *A*. *phagocytophilum* induction of degranulation increases the presence of PDI on the neutrophil surface, enhancing invasion. Alternatively, Asp1 engagement with PDI may play a role in stimulating neutrophil degranulation itself, as PDI binds various integrins and facilitates neutrophil activation [20]. Other pathogens such as *Helicobacter pylori* and *Peptoanaerobacter* spp. have also been shown to induce significant neutrophil degranulation, although the bacterial and host factors mediating the enhanced neutrophil degranulation are unknown [21,22].

## Outcomes of modulating neutrophil degranulation

Typically, microorganisms that inhibit neutrophil degranulation have greater survival following interactions with neutrophils. *N*. *gonorrhoeae* delays fusion of primary granules with the phagosome, allowing for increased intracellular bacterial survival [9]. This delay may allow *N*. *gonorrhoeae* to adapt to the neutrophil phagosome environment and persist within these cells [23]. Extracellular pathogens also inhibit neutrophil degranulation to promote survival. *Y*. *pestis* mutants that lack the type III secretion system effectors YopE and YopH (which are required to inhibit neutrophil primary granule release) are killed to a greater extent than wild-type *Y*. *pestis* during infection with isolated human neutrophils in vitro [12,24].

While inhibiting neutrophil degranulation allows for bacterial outgrowth, pathogens that induce neutrophil degranulation are frequently associated with severe pathology and host tissue damage that does not affect bacterial growth. For example, neutrophil elastase (contained within primary granules) contributes to lethality by damaging the lungs following intranasal infection with *Burkholderia thailandensis*, a model organism for studying melioidosis. Elastase-deficient mice inoculated with *B*. *thailandensis* survived intranasal challenge and all wild-type mice succumbed to the infection, and it was shown that elastase contributes to lung damage and vascular leakage. However, bacterial burdens were similar between the 2 mouse strains, indicating that neutrophil degranulation of elastase is harmful rather than protective during *B*. *thailandensis* infection [25]. Similarly, intravenous injection of the streptococcal M protein into mice is sufficient to induce neutrophil granule-mediated lung damage and vascular leakage, contributing to the development of acute lung injury [26]. Neutrophil degranulation worsens *Shigella* infection by augmenting its pathogenicity. Antimicrobial proteins released by degranulating neutrophils enhance *Shigella flexneri* adherence to and invasion of HeLa cells, likely by altering the surface charge of the bacteria to promote interactions with the host cell surface [27]. Thus, the inhibition of neutrophil degranulation can enhance bacterial survival by preventing deployment of antimicrobials contained within granules, whereas enhanced degranulation releases proteases that exacerbate infection through bystander damage of host tissues.

## Effects of tissue environment on neutrophil degranulation

Many studies that analyze neutrophil degranulation use freshly isolated neutrophils and a mono-infection tissue culture assay, and these experiments are critical to our understanding of neutrophil degranulation mechanisms. However, the local infection environment can impact neutrophil physiology and function. As such, it is also important to analyze neutrophil degranulation responses either in vivo or under conditions that more closely mimic the host environment. For instance, many infection sites are hypoxic. Hypoxia increases the magnitude of neutrophil degranulation responses for all granule types via signaling through PI3K [28]. Another complication of in vivo infection includes coinfecting organisms that alter immune responses. In a coculture system, neutrophils have greater degranulation responses when cultured with epithelial cells infected with respiratory syncytial virus (RSV) compared to mock-infected epithelial cells [29]. This enhanced degranulation could alter the capacity of neutrophils to respond to a secondary bacterial infection (a common complication of respiratory viral infection) due to changes in neutrophil function or viability following viral infection. Neutrophils also have a relatively short life span, making them susceptible to control by circadian rhythms. Neutrophils contain a cell-intrinsic program to reduce granule content during various times throughout the day, likely to minimize bystander tissue damage as neutrophils enter and exit various tissues [30]. Lastly, neutrophil responses to infection are typically studied as a whole population, such as measuring granule proteins in supernatants. However, recent imaging studies reveal disparate distributions of host proteins (such as calprotectin) among infectious abscesses even within the same organ [31]. This suggests that individual neutrophils can have varied degranulation responses throughout an infection, depending on a variety of host- and pathogen-dependent factors.

Targeting neutrophil degranulation is a successful strategy for several pathogens, resulting in enhanced disease either through greater bacterial growth or greater damage to host tissues (Fig 1). The conditions of the infection can also affect the capacity of neutrophils to degranulate in response to invading microorganisms and alter disease outcome. Understanding the mechanisms by which bacteria alter neutrophil degranulation to promote severe infection may reveal novel therapeutic targets for skewing the deleterious effects of neutrophils back toward the benefit of the host rather than the pathogen.
